# Grafting Modification of the Reactive Core-Shell Particles to Enhance the Toughening Ability of Polylactide

**DOI:** 10.3390/ma10080957

**Published:** 2017-08-16

**Authors:** Zhaokun Li, Shixin Song, Xuanchen Zhao, Xue Lv, Shulin Sun

**Affiliations:** Engineering Research Center of Synthetic Resin and Special Fiber, Ministry of Education, Changchun University of Technology, Changchun 130012, China; lzk540359560@163.com (Z.L.); 18743084020@163.com (S.S.); chenxin19921124@163.com (X.Z.)

**Keywords:** polylactide, toughening, core-shell particles, grafting modification

## Abstract

In order to overcome the brittleness of polylactide (PLA), reactive core-shell particles (RCS) with polybutadiene as core and methyl methacrylate-co-styrene-co-glycidyl methacrylate as shell were prepared to toughen PLA. Tert-dodecyl mercaptan (TDDM) was used as chain transfer agent to modify the grafting properties (such as grafting degree, shell thickness, internal and external grafting) of the core-shell particles. The introduction of TDDM decreased the grafting degree, shell thickness and the T_g_ of the core phase. When the content of TDDM was lower than 1.15%, the RCS particles dispersed in the PLA matrix uniformly—otherwise, agglomeration took place. The addition of RCS particles induced a higher cold crystallization temperature and a lower melting temperature of PLA which indicated the decreased crystallization ability of PLA. Dynamic mechanical analysis (DMA) results proved the good miscibility between PLA and the RCS particles and the increase of TDDM in RCS induced higher storage modulus of PLA/RCS blends. Suitable TDDM addition improved the toughening ability of RCS particles for PLA. In the present research, PLA/RCS-T4 (RCS-T4: the reactive core-shell particles with 0.76 wt % TDDM addition) blends displayed much better impact strength than other blends due to the easier cavitation/debonding ability and good dispersion morphology of the RCS-T4 particles. When the RCS-T4 content was 25 wt %, the impact strength of PLA/RCS-T4 blend reached 768 J/m, which was more than 25 times that of the pure PLA.

## 1. Introduction

As one kind of biobased and biodegradable thermoplastic polyester, polylactide (PLA) has drawn considerable attention during the past decade [1]. PLA can be prepared by condensation polymerization of lactic acid, which can be obtained from renewable agricultural resources such as corn, sugarcane and tapioca. This improves the possibility of replacing “fossil carbon” with “renewable carbon” and resolving the environmental pollution problem [2,3]. On the other hand, PLA shows excellent strength, modulus, biocompatibility and biodegradability, which is superior to that of some petroleum based polymers and performs useful application in automotive, package, medical and other industry fields [4,5,6,7,8,9]. However, like most thermoplastics, PLA also bears the inherent weaknesses of notch sensitivity and low notch impact toughness which limit the application of PLA in many areas [10,11,12,13,14].

In order to improve the toughness of PLA, some strategies such as plasticization [15,16,17,18], copolymerization [19,20,21] and blending with tough polymers and thermoplastic elastomers were widely adopted [22,23,24,25]. Core-shell structured elastomer particles are another important toughener of PLA [26,27,28,29,30,31,32,33,34]. As we know, the typical core-shell particles include an elastic rubber core phase, such as polybutadiene and poly (butyl acrylate), and a hard plastic shell phase, such as polystyrene and poly (methyl methacrylate). The core phase can cavitate and promote shear yielding of the matrix. The shell phase interacts with the matrix by physical or chemical coupling to ensure good dispersion of the particles in the blends. Li et al. [26] and Song et al. [27] prepared methyl methacrylate-co-butyl acrylate core-shell particles to toughen PLA. When the core-shell ratio was 79.2/20.8, the blend had a much higher impact strength of 77.1 kJ/m^2^. Glycidyl methacrylate-functionalized methyl methacrylate–butadiene (MB-g-GMA) was used by Hao to improve the toughness of PLA. The impact strength of the 75/25 PLA/MB-g-GMA blend was 59.5 kJ/m^2^, which was about 20 times that of pure PLA [30]. Sun prepared glycidyl methacrylate functionalized acrylonitrile-butadiene-styrene (ABS-g-GMA) particles to toughen PLA. Results showed that 1 wt % GMA was sufficient for the compatibilization and a notched impact strength of 540 J/m was achieved for the PLA/ABS-g-GMA blend [34].

Many structural factors can affect the toughening efficiency of the core-shell particles. These include core/shell composition, core/shell ratio, grafting degree, core particle size, crosslinking degree of the core, etc. Fortunately, all these factors can be controlled by the polymerization process. In the present paper, reactive core-shell particles (RCS) with polybutadiene (PB) as core and methyl methacrylate-co-styrene-co-glycidyl methacrylate as shell were prepared to toughen PLA. The PMMA components in the grafting shell have good miscibility with the PLA phase [35] and the epoxy groups of GMA can react with the carboxyl groups of PLA [22,23], which is beneficial for the improvement of compatibility. Tert-dodecyl mercaptan (TDDM) was used as a chain transfer agent to modify the grafting properties of the core-shell particles. The PLA blends with optimum toughness and stiffness balance were obtained by changing the grafting properties of the RCS particles with TDDM and a similar strategy can be used for other core-shell particle-toughened polymer blends.

## 2. Materials and Methods

### 2.1. Materials

PLA Grade 4032D was purchased from NatureWorks (Blair, NE, USA) in pellet form. The M_w_ of PLA was 207,000 with a polydispersity of 1.7. The RCS particles with different TDDM were prepared in our lab. The emulsion polymerization method was used to prepare the reactive core-shell particles (RCS) with polybutadiene (PB) as core and methyl methacrylate-co-styrene-co-glycidyl methacrylate as shell. The preparation method can been found in the reference [36]. The composition of the RCS particles is listed in Table 1.

### 2.2. Characterization of the RCS Particles

Particle size was measured by performing dynamic light scattering (DLS) on a Brookhaven 90 Plus laser particle analyzer (Brookhaven, New York, NY, USA). The shell thickness of the RCS particles was obtained from the particle size difference of the RCS particle and the PB core.

The grafting degree was determined by extracting the ungrafted copolymers and monomers using acetone. Acetone solutions of the dried RCS particles were shaken for 24 h at room temperature and then the solutions were centrifuged at 15,000 rpm in a GL-21M ultracentrifuge (Shanghai Centrifuge Co., Shanghai, China) for 30 min. The grafted particles were separated from the solution and the separation process was repeated three times. Then the separated RCS particles were dried in a vacuum oven at 60 °C for 12 h and weighed for the grafting degree calculation. The grafting degree is calculated from the following equation:

$$\mathrm{Grafting}\;\mathrm{degree}\;\left(\%\right)\;=100\times \frac{\mathrm{gel}\%-\mathrm{PB}\%}{\mathrm{PB}\%}$$

where gel% is the weight fraction of the acetone insoluble part in the sample and PB% is the weight fraction of polybutadiene in the core-shell particles.

### 2.3. Blending and Molding Procedures

The melt blending of the PLA/core-shell particles blends was performed on a Thermo Haake mixer (Thermo Scientific, Karlsruhe, Germany) at 50 rpm and 180 °C for 5 min. The content of core-shell particles in the PLA blends was 5 wt %, 10 wt %, 15 wt %, 20 wt % and 25 wt %. After blending, samples with different compositions were obtained by hot press molding for 5 min at 180 °C and cold press molding for 3 min at room temperature.

### 2.4. Mechanical Tests

The notched Izod impact strength was tested by an XJU-22 Izod impact tester (Chengde Tester Machinery Factory, Chengde, China) at 23 °C according to ASTM D256. The dimensions of each sample were 63.5 mm × 12.7 mm × 3.18 mm, with a notch depth of 2.54 mm. The tensile tests were performed with an Instron-3365 tensile tester (Instron, Boston, MA, USA) according to ASTM D638 at a crosshead speed of 50 mm/min at 23 °C.

### 2.5. Morphology Observation

Scanning Electron Microscopy (SEM) micrographs were characterized using a JSM6510 scanning electron microscope (JEOL, Tokyo, Japan). Before testing, all samples were coated with a gold layer for SEM observation with an operation voltage of 10 kV.

### 2.6. DMA Test

Dynamic mechanical analysis was carried out with the Diamond-DMA tester (Perkin Elmer, Yokohama, Japan) under tension mode in a temperature range from 30 to 180 °C. The heating rate was 3 °C/min and the frequency was 1 Hz. The dimension of the samples was 30 mm × 10 mm × 1 mm.

### 2.7. DSC Test

The melting and crystallization behavior of the samples were tested with a Perkin-Elmer DSC-7 (Perkin-Elmer, Waltham, MA, USA). The samples were heated from 30 to 200 °C at 10 °C/min under a nitrogen atmosphere to test the melting behavior and held for 3 min at 200 °C to remove the thermal history, then cooled at a rate of 10 °C/min to 30 °C to measure the crystallization properties. Two cycles were done for the test.

## 3. Results and Discussion

### 3.1. Properties of the Reactive Core-Shell Particles

The particle size of the tougheners is a very important factor in achieving the effective toughening. The optimum particle size is related to the nature of the matrix. Larger particles more than 1 μm are more effective when crazing is the principal mechanism of energy absorption. Small particles with a diameter of 200–400 nm work well if shear yielding dominates the toughening mechanism. In the present paper, the particle size of the PB core was 300 nm which is beneficial for the toughening of PLA.

**Figure 1:**
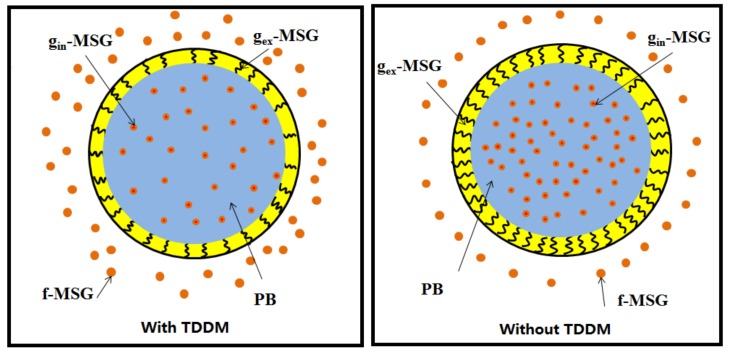
Schematic pictures of the RCS core-shell particles.

The grafting property—such as grafting degree, shell thickness, internal and external grafting—of the core-shell particles is also one important factor for the PLA toughening. As showed in Figure 1, the shell of the RCS particles is formed due to the grafting copolymerization of methyl methacrylate, styrene and glycidyl methacrylate monomers, which is called ‘external grafting’ (g_ex_-MSG). On the other hand, the swelling of the monomer into the PB particles can induce the grafting copolymerization inside the rubber particles which is called ‘internal grafting’ (g_in_-MSG). The external grafting of the shell phase can change the interfacial interaction between the particles and the PLA matrix, which affects the dispersion morphology and fracture properties of the toughened blends as discussed in the following parts.

**Figure 2:**
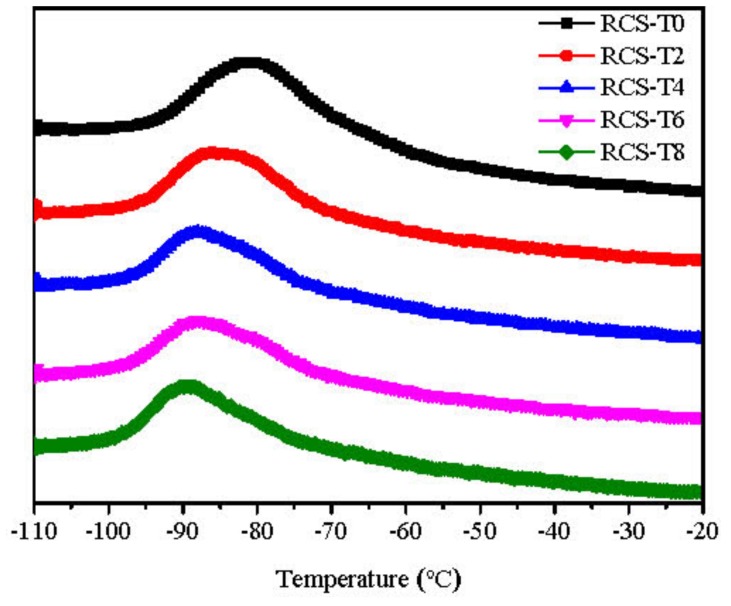
Tanδ-temperature curves of RCS particles with different TDDM content.

The introduction of TDDM can influence the glass transition temperature (T_g_) of the RCS particles. As a chain transfer agent, the addition of TDDM induces the propagating chain free radicals transfer to TDDM and decrease the ‘external grafting’ and ‘internal grafting.’ As shown in Figure 2, with the increase of TDDM content, the glass transition temperature (T_g_) of the PB phase shifts to lower the temperature. The lower T_g_ of the PB particle induces the increased cavitation ability of the PB core and improves the toughening ability of RCS particles as discussed in the following part.

**Figure 3:**
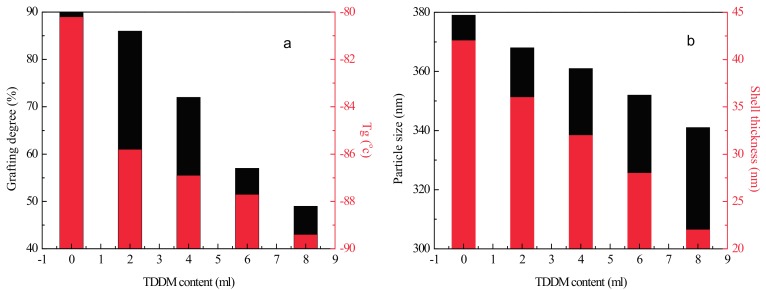
Influence of TDDM content on the properties of RCS particles (**a**) grafting degree and glass transition temperature (Tg) (**b**) particle size and shell thickness.

As it is shown in Figure 3a, an increase of TDDM content reduces the grafting degree and T_g_ of RCS particles. The particle size and shell thickness show a similar tendency to that presented in Figure 3b. From these results, we can conclude that TDDM decreases the grafting reactions of RCS particles, which lessens the restriction of stiff methyl methacrylate-co-styrene-co-glycidyl methacrylate (MSG)copolymers on the PB particles and reduces the T_g_ of the PB phase. The thinner shell reduces the interfacial strength between the RCS particles and the PLA matrix and affects the fracture process. On the other hand, the decrease of grafting degree leads to the increase of free methyl methacrylate-co-styrene-co-glycidyl methacrylate copolymers (f-MSG) as shown in Figure 1.

### 3.2. Dispersed Phase Morphology

**Figure 4:**
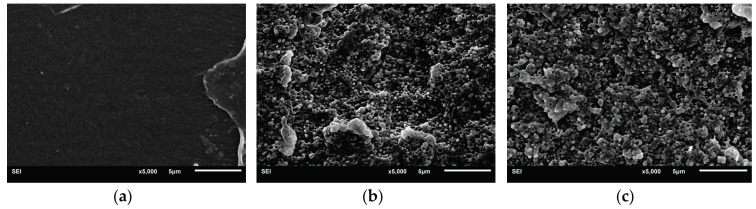

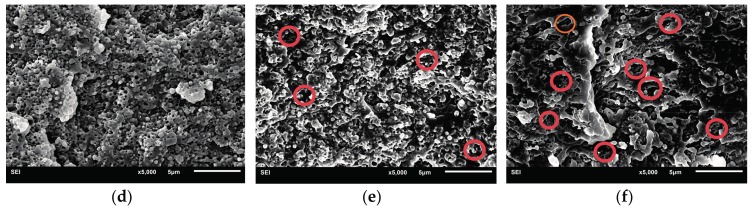
Dispersed phase morphology of PLA blends with different RCS particles (**a**) PLA; (**b**) PLA/RCS-T0; (**c**) PLA/RCS-T2; (**d**) PLA/RCS-T4; (**e**) PLA/RCS-T6; (**f**) PLA/RCS-T8. (The red cycles show the agglomeration of RCS particles)

The proper dispersion of the toughener is very important for polymer toughening. Usually, uniform dispersion of the dispersed phase is beneficial for improving the toughness of the materials. Since most of the polymers are not miscible with the tougheners, compatibilization strategies—such as reactive compabilization—are widely used to improve the compatibility between the polymers. In the present paper, the epoxy groups were introduced into the RCS core-shell particles by the glycidyl methacrylate component. The epoxy groups can react with the carboxyl or hydroxyl end groups of PLA, which improves the interfacial strength, reduces the interfacial tension, and inhibits the agglomeration of core-shell particles. Similar compatibilization reactions have been utilized in the PLA blends [19,20]. In Figure 4, it can be seen that the RCS-T0, RCS-T2 and RCS-T4 core-shell particles disperse in the PLA matrix uniformly. However, agglomeration takes place for the RCS-T6 and especially the RCS-T8 particles. The reason for the RCS particles’ agglomeration in these blends lies in the poor grafting, since the higher TDDM addition (≥6 mL) induces the lower grafting degree and thinner shell. So the core phase of the RCS particles cannot be covered well by the shell phase, which leads to the agglomeration of the RCS particles in the PLA matrix.

### 3.3. DSC Analysis

**Figure 5:**
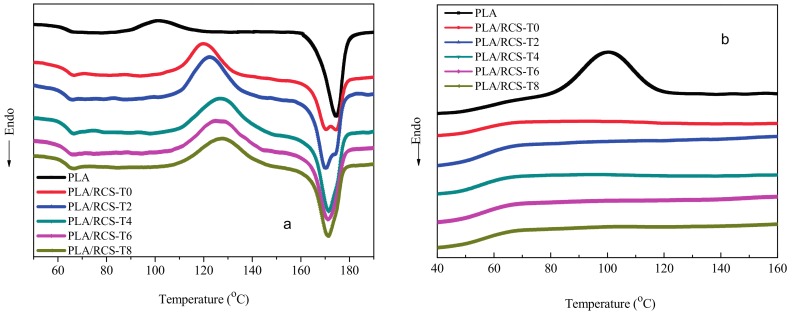
DSC thermogram of PLA and PLA/RCS blends recorded during the second heating (**a**) and cooling (**b**) runs.

The heating thermogram of the PLA and PLA/RCS blends during the second heating process is shown in Figure 5a. For the pure PLA, the cold crystallization and melting peaks can be found at 101.4 and 174.5 °C, respectively. The addition of RCS core-shell particles affects the melting and crystallization behavior significantly. In Figure 5a, the cold crystallization peak of PLA moves to a higher temperature with the increase of TDDM in the RCS core-shell particles. As for the PLA/RCS-T8 blend, the cold crystallization temperature reaches 127.6 °C. It is therefore demonstrated that the introduction of RCS particles reduces the crystallization ability of PLA, making PLA crystallization much more difficult. The RCS particles with much higher TDDM content induce more f-MSG, which can more easily react with the PLA and decrease its molecular segmental mobility, thus inhibiting its. crystallization process. On the other hand, the melting temperature (T_m_) of PLA shifts to a lower temperature with the addition of the RCS particles due to the imperfect crystallization.

The cooling thermogram of the PLA and PLA/RCS blends during the second cooling process is shown in Figure 5b. For the pure PLA, the crystallization peak occurs at 100.3 °C. However, no crystallization peaks can be found for the PLA/RCS blends, which further proves the inhibition effect of RCS particles on the crystallization ability of PLA. When the cooling rate is 10 °C/min, the PLA cannot crystallize. PLA crystallizes very slowly during the melt crystallization processes compared with other semicrystalline polymers, such as polypropylene (PP) and polyethylene (PE). When the cooling rate is fast, the PLA chains do not have enough time to form crystal structures and a much slower cooling rate is necessary in order to achieve the crystallization for PLA in the PLA/RCS blends.

### 3.4. Dynamic Mechanical Properties

**Figure 6:**
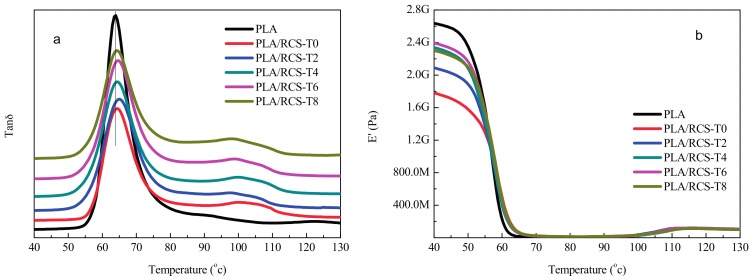
Tan δ-temperature (**a**) and E’-temperature (**b**) curves of PLA and PLA/RCS blends.

The dynamic mechanical properties of the PLA and PLA/RCS blends are shown in Figure 6. As can be seen from Figure 6a, PLA shows one T_g_ peak at 64 °C. With the addition of RCS particles into the PLA matrix, the T_g_ of PLA moves to higher temperature and the increase of T_g_ is no more than 1 °C. On the other hand, the T_g_ of methacrylate-co-styrene-co-glycidyl methacrylate copolymers between 100~110 °C occurs in the PLA/RCS blends, which shifts to a lower temperature with an increase of TDDM content in the RCS particles. The Tan δ-temperature curves further prove that PLA and RCS particles are not miscible since two separate T_g_ peaks exist. However, the PLA and RCS particles show good compatibility due to the compatibilization reactions between the carboxyl or hydroxyl end groups of PLA and the epoxy groups of the RCS particles, which induce the T_g_ peaks of PLA and methacrylate-co-styrene-co-glycidyl methacrylate copolymers to approach each other (the T_g_ increase of PLA is within 1 °C and the T_g_ decrease of methacrylate-co-styrene-co-glycidyl methacrylate copolymer is within 3 °C).

The E’-temperature curves of the PLA and PLA/RCS blends are shown in Figure 6b. It can be found that PLA shows higher storage modulus compared with the PLA/RCS blends due to the elastic nature of RCS particles. On the other hand, the PLA/RCS blends exhibit differing stiffness with changing levels of TDDM content in the RCS particles. The E’ of the PLA/RCS blends increases first with the varying of TDDM content and reaches the maximum for the PLA/RCS-T6 blend then decreases in some degree for the PLA/RCS-T8 blend. The difference between the E’ for the PLA/RCS blends lies in the different grafting properties of the RCS particles. Adding more TDDM leads to an increase of f-MSG, which can react with the PLA matrix and improve the stiffness of the blends. On the other hand, the lower grafting degree and shell thickness (such as RCS-T8) induce the serious agglomeration of the RCS particles in the PLA matrix, which forms defects during the deformation of the blends and induces the lower E’.

### 3.5. Mechanical Properties

**Figure 7:**
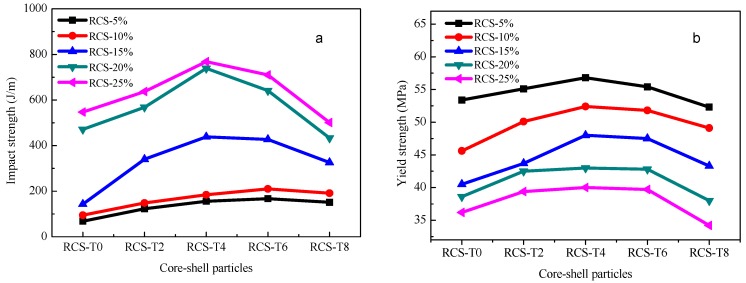
Mechanical properties of PLA/RCS blends (**a**) Impact strength; (**b**) Yield strength.

PLA is brittle and notch sensitive at room temperature and the notched impact strength of PLA is 30 J/m. The notched impact strength of PLA/RCS blends with different compositions is shown in Figure 7a. It can be found that RCS particles effectively improve the toughness of PLA. For all the RCS particles with different TDDM content levels, the impact strength of PLA/RCS blends increases with the RCS particle content. The brittle-ductile transition takes place between 15–20 wt % RCS-T0 content for the PLA/RCS-T0 blends while the brittle-ductile transition lies within the range of 10–15 wt % RCS content for the other PLA/RCS blends. So the introduction of TDDM into the RCS particles improves their toughening efficiency for PLA. On the other hand, RCS particles with different TDDM contents exhibit different toughening abilities. At the same RCS particles content, the impact strength of the blends increases with the TDDM content in the RCS particles, which reaches the maximum for PLA/RCS-T4 blends, then the impact strength deceases again for the PLA/RCS-T6 and PLA/RCS-T8 blends. When the RCS-T4 content is 25 wt %, the impact strength of PLA/RCS-T4 blends is 768 J/m which is more than 25 times that of the pure PLA.

The effect of RCS particles on the yield strength of PLA blends is shown in Figure 7b. For the pure PLA, the yield strength is 67 MPa. The yield strength of the PLA/RCS blends obviously decreases with the increase of RCS particle content, due to the elastomeric nature of the PB rubber phase in the RCS particles. On the other hand, the RCS particles with different TDDM content levels show a different influence on the yield strength of PLA blends. At the same RCS particle content, the yield strength of PLA/RCS blends increases with the TDDM content in RCS particles, which reaches the maximum for PLA/RCS-T4 and PLA/RCS-T6 blends before the yield strength deceases again for PLA/RCS-T8 blends. From the mechanical properties test we can conclude that RCS-T4 particles show optimum toughening ability and the blends display better toughness and stiffness balance.

### 3.6. Fracture Mechanism

**Figure 8:**
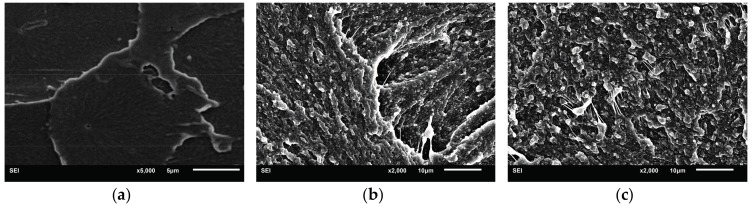

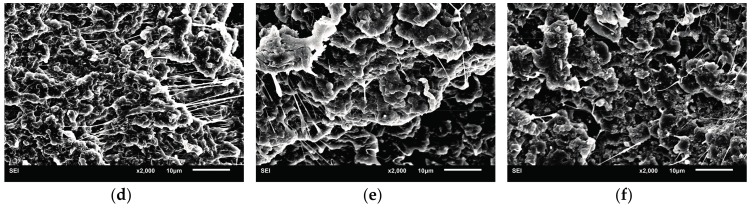
Fracture surface morphologies of PLA and PLA/RCS blends. (**a**) PLA; (**b**) PLA/RCS-T0; (**c**) PLA/-T2; (**d**) PLA/RCS-T4; (**e**) PLA/RCS-T6; (**f**) PLA/RCS-T8.

The notched impact fracture surface of PLA and PLA/RCS blends is observed by SEM. The fracture surface morphology of pure PLA is shown in Figure 8a. As expected, most areas of the PLA fracture surface is smooth and no stress whitening and plastic deformation can be found, which indicates the absence of an effective energy absorption-related deformation process. PLA fractures in a brittle way and shows a much lower impact strength (30 J/m). Different from the fracture morphology of PLA, PLA/RCS blends show obvious stress whitening. Figure 8b–f display many root-like whiskers and fibril-like structures on the fracture surface, which indicate that shear yielding of the PLA matrix has taken place and the materials fracture in a ductile way. Compared with other blends, the PLA/RCS-T4 blend shows more prominent plastic deformation which is consistent with its higher impact strength.

**Figure 9:**
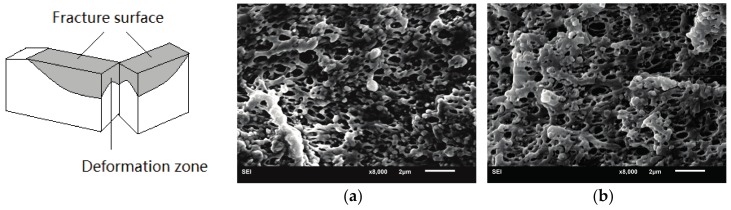

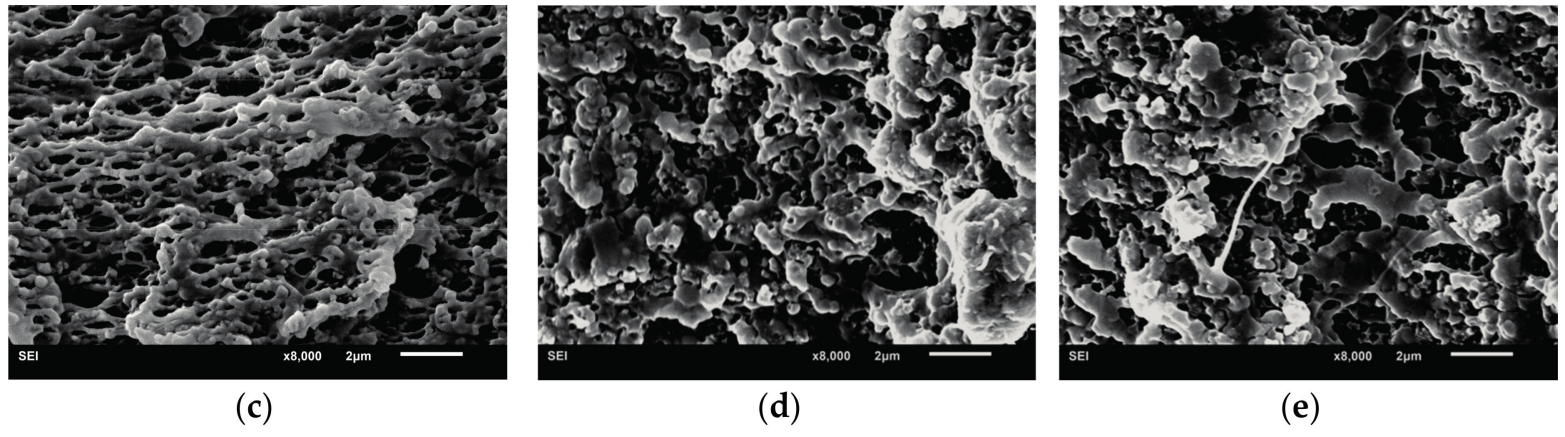
Deformation zone morphologies of PLA/RCS blends. (**a**) PLA/RCS-T0; (**b**) PLA/RCS-T2; (**c**) PLA/RCS-T4; (**d**) PLA/RCS-T6; (**e**) PLA/RCS-T8.

In order to correlate the external fracture surface morphology with the internal deformation mechanisms in the stress whitening zone, the morphology inside the deformation zone—as shown in Figure 9—was observed by SEM. For rubber toughened pseudoductile polymers, voiding formation is very important for achieving plastic deformation. Cavitation of the rubber particles and debonding between the rubber particles and the matrix can lead to the generation of voids during the deformation process. The effect of the voids is to relieve the hydrostatic stress and promote extensive shear yielding of the matrix. The formation of the voids is related to the cavitation resistance of the modifiers and the interfacial strength between the modifiers and the matrix [37]. In the present research, the introduction of TDDM changes the grafting properties of the RCS particles, which can affect the formation of the voids. On the one hand, the addition of TDDM decreases the grafting degree and T_g_ of PB particles which help to improve the cavitaton ability of the RCS particles. On the other hand, the decreased grafting degree and shell thickness reduce the interfacial strength which are in favor of the debonding. From Figure 9a–c, therefore, it can be found that many voids exist in the deformation zone due to the cavitation of the PB particles and debonding between RCS particles and the PLA matrix. At the same time, extensive shear yielding occurs for the blends. Compared with PLA/RCS-T0 and PLA/RCS-T2 blends, PLA/RCS-T4 blend shows bigger voids size and more significant plastic deformation, which are related to its grafting properties due to the higher TDDM content. However, higher TDDM content in RCS particles induces the agglomeration of the particles, which induces some big voids in the deformation zone of PLA/RCS-T6 and PLA/RCS-T8 blends. The big voids can form deficiency during the deformation process and decrease the toughening efficiency of the RCS particles. From the SEM microscopy of the fracture surface and deformation zone, it can therefore be concluded that cavitation and debonding coexist in the deformation process, promoting the shield yielding of the PLA matrix and inducing the toughness improvement of the PLA/RCS blends.

## 4. Conclusions

The reactive core-shell particles (RCS) with polybutadiene as core and methyl methacrylate-co-styrene-co-glycidyl methacrylate as shell were effective at toughening PLA. Modification of the grafting properties of the RCS particles using TDDM provides a strategy for preparing core–shell particles for superior toughening of PLA. The introduction of TDDM affected the grafting degree, shell thickness, T_g_ and dispersion morphology of the RCS particles which influenced the final mechanical properties of the PLA/RCS blends. Deformation results proved that cavitation/debonding of the RCS particles and shear yielding of the PLA matrix were the major toughening mechanisms. The addition of TDDM resulted in lower grafting degree and T_g_ of PB particles, which improved their cavitation/debonding ability. Suitable TDDM content promoted the toughening efficiency of the RCS particles and induced a lower brittle–ductile transition for the PLA/RCS blends. The higher TDDM content led to a lower grafting degree and shell thickness which induced agglomeration of the RCS particles and reduced the mechanical properties of the blends. The PLA blends with optimum toughness and stiffness balance were obtained by changing the grafting properties of the RCS particles using TDDM and a similar strategy could be used for other core–shell particle-toughened polymer blends.

## Figures and Tables

**Table 1:** Composition of the reactive core shell (RCS) particles used in the paper.

Designation Used Here	Polybutadiene (PB) Content (wt %)	Styrene/Methyl Methacrylate (St/MMA) (wt/wt)	Glycidyl Methacrylate (GMA) Content (wt %)	Tert-Dodecyl Mercaptan (TDDM) Content (mL/wt %)
RCS-T0	50	3/1	1	0/0
RCS-T2	50	3/1	1	2/0.38
RCS-T4	50	3/1	1	4/0.76
RCS-T6	50	3/1	1	6/1.15
RCS-T8	50	3/1	1	8/1.53

## References

[1] Slomkowski S., Penczek S., Duda A. (2014). Polylactides—An overview. Polym. Adv. Technol..

[2] Nagarajan V., Mohanty A.K., Misra M. (2016). Perspective on polylactic acid (PLA) based sustainable materials for durable applications: Focus on toughness and heat resistance. ACS Sustain. Chem. Eng..

[3] Muiruri J.K., Liu S.L., Teo W.S., Kong J., He C. (2017). Highly biodegradable and tough polylactic acid–cellulose nanocrystal composite. ACS Sustain. Chem. Eng..

[4] Gao H.H., Qiang T. (2017). Fracture Surface Morphology and impact strength of cellulose/PLA composites. Materials.

[5] Balakrishnan H., Hassan A., Imran M., Wahit M.U. (2012). Toughening of Polylactic Acid Nanocomposites: A Short Review. Polym.-Plast. Technol. Eng..

[6] Sun Y., He C.B. (2013). Synthesis, stereocomplex crystallization, morphology and mechanical property of poly(lactide)-carbon nanotube nanocomposites. RSC Adv..

[7] Sun Y., He C. (2013). Biodegradable “core-shell” rubber nanoparticles and their toughening of poly (lactides). Macromolecules.

[8] Bai H., Bai D., Xiu H., Liu H., Zhang Q., Wang K., Deng H., Chen F., Fu Q., Chiu F.-C. (2014). Towards high-performance poly (Llactide)/elastomer blends with tunable interfacial adhesion and matrix crystallization via constructing stereocomplex crystallites at the interface. RSC Adv..

[9] Brzeziński M., Biela T. (2014). Polylactide nanocomposites with functionalized carbon nanotubes and their stereocomplexes: A focused review. Mater. Lett..

[10] Raquez J.-M., Habibi Y., Murariu M., Dubois P. (2013). Polylactide (PLA)-based nanocomposites. Prog. Polym. Sci..

[11] Zeng J., Li K., Du A. (2015). Compatibilization strategies in poly(lacticacid)-based blends. RSC Adv..

[12] Liu H., Zhang J. (2011). Research progress in toughening modification of poly(lactic acid). J. Polym. Sci. Part B: Polym. Phys..

[13] Anderson K.S., Schreck K.M., Hillmyer M.A. (2008). Toughening polylactide. Polym. Rev..

[14] Rasal R.M., Janorkar A.V., Hirt D.E. (2010). Poly(lactic acid) modifications. Prog. Polym. Sci..

[15] Kulinski Z., Piorkowska E. (2005). Crystallization, structure and properties of plasticized poly(l-lactide). Polymer.

[16] Ozkoc G., Kemaloglu S. (2009). Morphology, biodegradability, mechanical, and thermal properties of nanocomposite films based on PLA and plasticized PLA. J. Appl. Polym. Sci..

[17] Kulinski Z., Piorkowska E., Gadzinowska A.K., Stasiak M. (2006). Plasticization of Poly(l-lactide) with Poly(propylene glycol). Biomacromolecules.

[18] Ge H.H., Yang F., Hao Y.P., Wu G.F., Zhang H.L., Dong L.S. (2013). Thermal, Mechanical, and Rheological Properties of Plasticized Poly(L-lactic acid). J. Appl. Polym. Sci..

[19] Odent J., Leclere P., Raquez J., Dubois P. (2013). Toughening of polylactide by tailoring phase-morphology with P [CL-co-LA] random copolyesters as biodegradable impact modifiers. Eur. Polym. J..

[20] Odent J., Raquez J., Duquesne E., Dubois P. (2012). Random aliphatic copolyesters as new biodegradable impact modifiers for polylactide materials. Eur. Polym. J..

[21] Peponi L., Navarro-Baena I., Báez J.E., Kenny J.M., Marcos-Fernández A. (2012). Effect of the molecular weight on the crystallinity of PCL-b-PLLA diblock copolymers. Polymer.

[22] Oyama H.T. (2009). Super-tough poly(lactic acid) materials: Reactive blending with ethylene copolymer. Polymer.

[23] Su Z., Li Q., Liu Y., Hu G., Wu C. (2009). Compatibility and phase structure of binary blends of poly(lactic acid) and glycidyl methacrylate grafted poly(ethylene octane). Eur. Polym. J..

[24] Kang H., Hu X., Li M., Zhang L., Wu Y., Ning N., Tian M. (2015). Novel biobased thermoplastic elastomer consisting of synthetic polyester elastomer and polylactide by in situ dynamical crosslinking method. RSC Adv..

[25] Ojijo V., Ray S.S., Sadiku R. (2013). Toughening of biodegradable polylactide/poly(butylene succinate-co-adipate) blends via in situ reactive compatibilization. ACS Appl. Mater. Interfaces.

[26] Li W., Zhang Y., Wu D.D., Li Z.L., Zhang H.L., Dong L.S., Sun S.L., Deng Y.J., Zhang H.X. (2015). The effect of core-shell ratio of acrylic impact modifier on toughening PLA. Adv. Polym. Technol..

[27] Song X., Chen Y., Xu Y., Wang C. (2014). Study on Tough Blends of Polylactide and Acrylic Impact Modifier. BioResources.

[28] Zhang H.L., Liu N.N., Ran X.H., Han C.Y., Han L.J., Zhang Y.G., Dong L.S. (2012). Toughening of polylactide by melt with methyl methacrylate-butadiene-styrene copolymer. J. Appl. Polym. Sci..

[29] Liang H.Y., Hao Y.P., Bian J.J., Zhang H.L., Dong L.S., Zhang H.X. (2015). Assessment of miscibility, crystallization behavior, and toughening mechanism of polylactide/acrylate copolymer blends. Polym. Eng. Sci..

[30] Hao Y.P., Liang H.Y., Bian J.J., Sun S.L., Zhang H.L., Dong L.S. (2013). Toughening of polylactide with epoxy-functionalized methyl methacrylate-butadiene copolymer. Polym. Int..

[31] Zhang H.L., Liang H.Y., Bian J.J., Hao Y.P., Han L.J., Wang X.M., Zhang G.B., Liu S.R., Dong L.S. (2014). Influence of acrylic impact modifier on plasticized polylactide blown films. Polym. Int..

[32] Li W., Wu D.D., Sun S.L., Wu G.F., Zhang H.X., Deng Y.J., Zhang H.L., Dong L.S. (2014). Toughening of polylactide with epoxy-functionalized methyl methacrylate-butyl acrylate copolymer. Polym. Bull..

[33] Wu N.J., Zhang H. (2015). Toughening of poly(L-lactide) modified by a small amount of acrylonitrile-butadiene-styrene core-shell polymer. J. Appl. Polym. Sci..

[34] Sun S., Zhang M., Zhang H., Zhang X. (2011). Polylactide toughening with epoxy-functionalized grafted acrylonitrile−butadiene−styrene particles. J. Appl. Polym. Sci..

[35] Zhang G., Zhang J., Wang S., Shen D. (2003). Miscibility and phase structure of binary blends of polylactide and poly(methyl methacrylate). J. Polym. Sci. Part B: Polym. Phys..

[36] Guo Y., Sun S., Zhang H. (2014). Modification of the core-shell ratio to prepare PB-g-(MMA-co-St-co-GMA) particle-toughened poly(butylene terephthalate) and polycarbonate blends with balanced stiffness and toughness. RSC. Adv..

[37] Pawlak A., Galeski A., Rozanski A. (2014). Cavitation during deformation of semicrystalline polymers. Prog. Polym. Sci..

